# Associations between Chronotype, Adherence to the Mediterranean Diet and Sexual Opinion among University Students

**DOI:** 10.3390/nu12061900

**Published:** 2020-06-26

**Authors:** Pedro Manuel Rodríguez-Muñoz, Juan Manuel Carmona-Torres, Cristina Rivera-Picón, Fabio Fabbian, Roberto Manfredini, María Aurora Rodríguez-Borrego, Pablo Jesús López-Soto

**Affiliations:** 1 Department of Nursing, Instituto Maimónides de Investigación Biomédica de Córdoba, 14005 Córdoba, Spain; juanmanuel.carmona@uclm.es (J.M.C.-T.); en1robom@uco.es (M.A.R.-B.); n82losop@uco.es (P.J.L.-S.); 2 Faculty of Health Sciences, Nursing. Universidad Pontificia de Salamanca, 37002 Salamanca, Spain; criverapi@upsa.es; 3 Facultad de Fisioterapia y Enfermería, Universidad de Castilla-La Mancha, 45071 Toledo, Spain; 4 Faculty of Medicine, Surgery and Prevention, University of Ferrara, 44121 Ferrara, Italy; f.fabbian@ospfe.it (F.F.); mfr@unife.it (R.M.); 5 Department of Nursing, Universidad de Córdoba, 14004 Córdoba, Spain; 6 Hospital Universitario Reina Sofía de Córdoba, 14004 Córdoba, Spain

**Keywords:** Mediterranean diet, chronotype, sexual opinion, university students

## Abstract

A person’s chronotype determines different habits, among which are eating and physical activity. Furthermore, at the university stage, social and organisational factors have a direct effect on students’ daily attitudes and habits. Adherence to the Mediterranean diet is linked to better sleep quality and less social jet lag, but association with chronotype or sexual opinion remains unclear. The aim of this study was to assess the associations between chronotype, adherence to the Mediterranean Diet, and sexual opinion. A multicentre observational study enrolled 457 students, from the University of Castilla-La Mancha and the University of Cordoba. Sociodemographic data and adherence to the Mediterranean diet, chronotype, physical activity, and sexual opinion were collected with validated questionnaires. The study period was from December 2017 to January 2018. Our results reported that students with an evening chronotype (E-type), with evening preferences, had a lower adherence to the Mediterranean diet and showed a higher tendency towards erotophilia. E-type students reported a significantly lower intake of fruits, vegetables, pulses, cereals, and olive oil, and higher breakfast skipping. Therefore, among the measures to promote healthy habits (obesity prevention, sexual education, socialisation, etc.), chronotype and an analysis of the impact of the schedules established by the universities must be considered.

## 1. Introduction

The university stage is a period where habits can be altered by organisational and socio-cultural factors, which can have a modifying effect on chronotype: a variable that can modulate different habits such as diet, physical activity, or sexual opinion. Likewise, the university stage is a period of critical education for the development of dietary habits which determine future health [1,2]. On the other hand, health behaviours during college years may remain throughout adulthood [3]. Chronobiology is a scientific discipline that is based on the study of biological rhythms produced in living organisms at their different levels of organisation [4]. Circadian rhythms are the best-known in humans. These are biological rhythms with a duration of around 24 h (20 to 28 h), which regulate the changes that can occur in physical and mental characteristics throughout the day [4,5]. 

In turn, the individual differences with respect to the sleep–wake pattern, the so-called chronotype, is one of the factors that significantly influence the functioning of the organism and the behaviour of both adolescents and young adults [6,7]. There are people with morning preferences, morning chronotype (M-type), who fall asleep early, get up early, and perform activities better in the early hours of the day. At the other extreme are people with evening preferences, evening chronotype (E-type), who choose both a later bedtime and wake-up time, and who perform better in the afternoon or evening [6]. 

Young people are characterised by the risk of being predisposed to obesity, a decrease in healthy diet and physical activity [8], as well as by having more E-type, a shorter duration of sleep on weekdays, and sleeping later and for a longer duration on weekends [6]. They also experience more sleepiness during the day and have more sleep-related problems compared with other chronotypes [6,9]. Furthermore, young people with an E-type seem to have a higher risk of depressed mood, suicidal thoughts, and lower self-esteem [6,10,11].

An association has been observed between the academic activity of students and the different chronotypes [12]. University students with an (M-type) have better grades than university students with E-types [12]. This can be justified by the fact that E-type students usually have more sleep deprivation than M-type ones, and because the lesson timetable is usually scheduled in the early morning [12]. Many students with an E-type report feeling sleepy during the day [6]. Lack of sleep, poor sleep quality, and irregular bedtime schedules could largely justify this feeling of sleepiness [6].

In turn, E-type is related to a greater use of substances such as caffeine, alcohol, tobacco and marijuana [3], and lower academic performance [7]. Likewise, an association between chronotype and nutrition has been observed, relating the E-type with a less healthy diet than the M-type [13]. 

From a general point of view, in Spain the usual eating pattern is the Mediterranean diet, which is recognised as a healthy diet [14]. The Mediterranean diet is characterised by a high consumption of fruits and vegetables, as well as legumes and cereals, the use of olive oil as the main lipid, regular consumption of fish, and the use of techniques to boil food in water, and frying only with olive oil. This diet entails a low meat intake, predominantly poultry, a moderate-low but regular intake of dairy products, the consumption of only simple carbohydrates, and low or no intake of industrially processed foods [14,15].

Regarding nutrition, university students are quite a vulnerable group [2]. The university period is the time when most students are taking responsibility for the first time for their diet. They often minimise or skip some meals, prefer to eat fast food, snack between meals, and consume alcohol frequently. The diet they typically follow is high in fat and low in dietary fibre. In general, university is an important stage of education for the development of eating habits and future health [2]. 

Eating habits are determined by knowledge of nutrition and food—the more information, the greater the healthy eating habits. However, social, cultural, and economic factors, and food preferences influence diet [2,16].

Another aspect to consider is sexual opinion. Attitudes towards sexuality are related to morning or evening preferences [17,18]. The erotophobia–erotophilia dimension, both of which are a learned disposition to respond to sexual stimuli, is used to measure sexual opinion. Erotophobia entails the response with more negative attitudes towards the sexual stimulus and feelings of avoidance, and erotophilia is an opinion contrary to erotophobia, a positive attitude towards the sexual stimulus, with more favourable emotions and evaluations leading to a greater search for sexual stimuli [17,18,19]. There seems to be a higher evening peak of sexual activity and desire [20]. Some authors report that E-types are most likely to be affected because sexual behaviour happens at their optimal time of day [11,20]. 

To our knowledge, there is little evidence on the effect of chronotype on adherence to the Mediterranean diet and sexual opinion. We hypothesised that students with a tendency to the E-type would exhibit less adherence to the Mediterranean diet and a disposition to respond to sexual cues along a positive dimension of affect and evaluation (erotophilia).

## 2. Materials and Methods 

### 2.1. Design and Participants

University students, from the Universities of Cordoba (UCO) and Castilla-La Mancha (UCLM) in Spain, were enrolled in a multicentre observational descriptive study. As inclusion criteria, all students (undergraduate, master’s, and doctoral) enrolled in both universities were considered (total sampling). 

### 2.2. Variables

Sociodemographic variables of the student (age, sex, university, university degree, academic year, place of residence, marital status) and variables related to parents’ unhealthy habits (history of alcohol use, history of tobacco use, history of illness related to tobacco/alcohol consumption, history of physical activity) were collected. Self-reported students’ height (in cm) and weight (in kg) were reported. Using these data, self-reported body mass indexes (BMI) were calculated. 

Further, with respect to the student, adherence to the Mediterranean diet, chronotype, physical activity, and sexual opinion were collected

### 2.3. Data Collection

The data collection was carried out from 8 December 2017 to 31 January 2018. An online questionnaire using the Google Forms system (Google Inc., Mountain View, CA, USA), which is a free tool and uses a user-friendly web interface, was prepared to ensure the confidentiality of the users. At the top of the form, an information and informed consent sheet was included. By email, the information and link to the questionnaire were sent to the people in charge of the dissemination of the study at both universities. 

### 2.4. Adherence to the Mediterranean Diet

The Mediterranean diet quality index for children and adolescents (KIDMED) [21] consists of 16 questions answered dichotomously (yes/no). Twelve questions represent a positive aspect in relation to the Mediterranean diet, and 4 represent a negative one. The affirmative answers to the questions that refer to a positive aspect add 1 point, and the affirmative answers to the questions with a negative connotation subtract one point. The total score results in a classification into three categories: (i) optimal Mediterranean diet (high adherence): score of 8 to 12 points; (ii) need to improve the eating pattern to adapt it to the Mediterranean diet (medium adherence): score of 4 to 7 points; and (iii) very low-quality Mediterranean diet (low adherence): score from 0 to 3 points.

### 2.5. Chronotype

We used a reduced scale of Horne and Östberg’s morningness–eveningness questionnaire (rMEQ) [22], which is a validated scale adapted and standardised for the Spanish student population [23]. This reduced version resolves issues related to Horne and Ostberg’s questionnaire. Some of the 19 items in the initial version collect information concerning other dimensions beyond the morning–evening dimension [23]. 

rMEQ consists of 5 items which have 5 response options except for items 2 and 5, which have only 4. The score ranges from 4 to 25 points. It distinguishes scores from 4 to 7 points as clearly evening, from 8 to 11 points as moderately evening, from 12 to 17 points as no type (intermediate chronotype), from 18 to 21 points as moderately morning, and from 22 to 25 points as clearly morning.

### 2.6. Sexual Opinion

The Revised Sexual Opinion Survey (R-SOS) [18] was used to collect sexual opinions. This questionnaire measures the erotophobia–erotophilia dimension, defined as the willingness to respond to sexual stimuli, ranging from a negative point, erotophobia, to a positive point, erotophilia. It consists of 20 items, direct and inverse, which are answered on a Likert-type scale, ranging from 1 (strongly disagree) to 7 (strongly agree). The total score ranges from 0, the highest level of erotophobia, to 120, the highest level of erotophilia.

### 2.7. Physical Activity 

To evaluate physical activity, subjects completed the short version of the International Physical Activity Questionnaire-Short Form (IPAQ-SF) [24], validated in the general Spanish population [25] and Spanish students [26]. The IPAQ measures three characteristics of physical activity: frequency (measured in days per week), duration (time per day), and the metabolic equivalent of task (MET). The first two characteristics are established by direct questions. Regarding MET, reference values are: (i) walking: 3.3 MET; (ii) moderate physical activity: 4 MET; and (iii) vigorous activity: 8 MET. The total physical activity score is classified according to three categories: low, medium, or high.

### 2.8. Statistical Analysis

Data analysis was performed using the statistical program International Business Machines (IBM) Statistics Package for the Social Sciences (SPSS) version 23 (IBM Corp, Armonk, NY, USA). Descriptive characteristics for continuous variables (age, BMI, KIDMED score, chronotype score, sexual opinion score, and physical activity) as well as the mean and standard deviation (SD) were calculated. Percentages (%) were used for categorical variables (sex, chronotype, BMI, KIDMED index). A comparison of proportions of the categorical variables was also performed using chi-square or Fisher’s tests for the contingency tables. To compare means of chronotypes and KIDMED test items, a student’s t-test was used. Analysis of variance (ANOVA) was also performed to analyse the differences between two or more means. Pearson correlations were used to establish associations between chronotype and sexual opinion with adherence to the Mediterranean diet and BMI. Adjusted linear regression analyses were carried out when any significant association existed. In all statistical tests, testing was significant when *p* < 0.05. 

### 2.9. Ethical Considerations 

The study was authorised by the Foundation for Biomedical Research of Córdoba (FIBICO) and by the Universities of Córdoba and Castilla-La Mancha. Likewise, it received a favourable report from the Research Ethics Committee of the Province of Córdoba (Act N. 269, ref. 3640).

## 3. Results

### 3.1. Participants’ Characteristics, Adherence to the Mediterranean Diet, Chronotype, Physical Activity, and Erotophobia–Erotophilia

A total of 464 students participated in the study, of whom 7 were excluded due to incomplete questionnaires. Finally, the sample consisted of 457 students. Of these students, 33.3% were men and 66.7% women. The mean age was 20.93 years (SD = 3.28), and 36.5% studied at UCO and 63.5% at UCLM. With respect to the university studies, the majority (93.4%) were undergraduate students. There was greater participation on the part of students in the health sciences (29.3%), sciences (24.1%), and social and legal sciences (21.7%). Participants’ characteristics according to adherence to the Mediterranean diet are shown in Table 1.

Most students were single (96.1%). A total of 44.6% cohabited with their parents/tutors and 43.1% with other students in rental apartments. Half of the students exhibited a vigorous level of activity. The majority of students had protected sex (91.9%) and had an average score in the erotophobia–erotophilia dimension, with most tending towards erotophilia (77.9 ± 19.9). Men show a higher mean of erotophilia (82.6 ± 18.3) than women (75.4. ± 20.3): UCO students scored with higher values (78.1 ± 19) than UCLM ones (77.8 ± 20.4). Regarding residence, those students who cohabit with peers have a higher level in low adherence to the Mediterranean diet (58.3%) than those who cohabit with parents (34.7%). About two thirds of the students did not have a defined chronotype. Students with an E-type were more common (3.9% extreme and 16.8% moderate) than those with a morning chronotype (M-type) (15.5% moderate and 1.1% extreme). Males had more defined chronotypes than females (more frequently in E-type and M-type). In relation to the students’ relatives, there was a higher frequency of parents who did not consume alcohol (72%) but did smoke (65.6%), and 7.2% had a history of tobacco/alcohol-related illnesses. More than half of parents did not engage in a regular physical sport. 

Table 2 shows adherence to the Mediterranean diet among the population studied considering chronotype. About two thirds of the students (63%) had poor or medium adherence to the Mediterranean diet. One third of students go to a fast-food restaurant more than once a week, and a significant number of students (16.2%) skip breakfast. In relation to gender, no significant differences were found for the KIDMED index and for the majority of items, except for “use of olive oil at home” and “take two yogurts and/or some cheese (40 g) daily”, which were significantly more frequent in males. University students whose parents engage in physical exercise have a lower level in low adherence to the Mediterranean diet than those who do not (10.6% versus 20.2%; *p* = 0.02).

### 3.2. Tendency to E-Type is Associated with a Lower Adherence to the Mediterranean Diet and Erotophilia

Students with lower values in chronotype (E-type) showed a lower adherence to the Mediterranean diet and a higher tendency towards erotophilia. Although no significance was detected, higher values in BMI tended towards erotophilia (Table 3).

### 3.3. E-Type is Associated with a Lower Intake of Fruit, Vegetables, Pulses, Cereals, Olive Oil and a Higher Incidence of Skipping Breakfast 

Considering chronotype as a continuous variable, E-type students indicated that they did not take fruit every day or a second serving, did not consume vegetables once or more than once a day, did not like pulses and did not eat them more than once a week, and did not consume cereals or cereal products (bread) for breakfast (*p* < 0.05). In addition, students who skipped breakfast were E-types (*p* < 0.001).

## 4. Discussion 

In the present study, it has been observed how E-type personality was associated with lower adherence to the Mediterranean diet and a tendency towards erotophilia. Likewise, chronotype has been shown to be an important variable that interferes in two highly relevant areas at the university stage, diet, and sexual opinion.

Compared with other studies, university students in this study had higher adherence to the Mediterranean diet than other investigations carried out among university students in Castilla-La Mancha [27,28,29], which ranged from 5 to 9%. University students from Navarra also showed less adherence [30] as did studies from other countries, such as Cyprus [31], Turkey [32], and Tunisia [33]. On the other hand, similar data are shown in Spanish universities in Madrid [34], Murcia [35], and Barcelona [36]. For their part, Zurita-Ortega et al. [37] demonstrated even superior data regarding high adherence to the Mediterranean diet in university students in Granada, Ceuta, and Melilla, with 78% having high adherence.

One third of students go to a fast-food restaurant more than once a week. This fact may be influenced by time pressures at university, ease and convenience, and a lack of cooking knowledge [38]. Furthermore, Oexle et al. [39] claim that fast-food consumption is due to the availability of this type of food in a person’s immediate area or neighbourhood. 

A total of 16.2% of university students skip breakfast, the eating of which is an important habit to maintain an ideal weight [40]. For students who are still growing and developing, breakfast is important for both physiological requirements and mental functions [41]. Skipping breakfast is associated with a higher fat intake, a lower intake of proteins, vitamins, and minerals [42], and, consequently, a higher BMI, which is associated with greater levels of being overweight and obesity [42,43].

Regarding chronotype, by sex, males are more E-type than women, with 24.3% and 19%, respectively. This was also confirmed by other studies involving university students in the United States [7], Spain [44], and the general population in Brazil [45]. On the other hand, there are studies where eveningness and sleep problems are related, especially in women who have poor sleep quality, which presents itself in the form of insomnia and nightmares [46,47]. Eveningness may impact general health, either physical or mental, especially in women [46]. Eveningness related to higher hostility, with men scoring higher than women in physical and verbal aggression [48]. Regarding age, from persons’ birth to their twenties, eveningness increases, after which chronotype tends towards more morning [49,50]. Likewise, social lifestyle, occupation, and school time schedules may also play a role in determining chronotype [51]. Chronotype is associated with psychological and psychopathological issues, E-type is associated with impulsivity and anger, depression, anxiety disorders, and nightmares [46]. 

From the analysis carried out, it was found that students with the M-type are those with the highest level of adherence to the Mediterranean diet. In contrast, the E-type shows the lowest adherence to the Mediterranean diet. Therefore, there is an association between M-type and high adherence to the Mediterranean diet, and E-type and low adherence to the Mediterranean diet, as noted by Muscogiuri et al. and Zerón-Rugerio et al. [36,52].

Studies focusing on adherence to the Mediterranean diet, quality and duration of sleep, and insomnia in older adults have been carried out [53,54,55,56]. These authors point out improvements in sleep quality, duration, and reduced insomnia with high adherence to the Mediterranean diet. Gianfredi et al. [57] compared sleep quality with adherence to the Mediterranean diet among university students in Italy, with results similar to those found in the aforementioned studies. It can be thought that the association with the quality of sleep may be due to the foods included in the Mediterranean diet. These foods are fruits and vegetables, legumes, nuts, fish, white meat, and foods rich in tryptophan (Trp), an essential amino acid only provided by the diet, which has positive effects on the induction and maintenance of sleep [57,58]. Another fact to keep in mind is that the Mediterranean diet is characterised by the importance given to breakfast, a meal that students with an E-type often skip [2,36,43,59], as shown in the present study.

An important body of research shows that chronotype may be associated with psychological and psychopathological issues [46]. For example, morningness has been associated with a reduced probability of smoking and drinking alcohol [60], while E-type subjects take more risks with respect to decisions related to finances, and the ethical and recreational spheres [61]. Greater impulsivity and risk-taking have been linked to unrestricted sociosexual orientation (individual differences in willingness to engage in noncommitted sexual relations) [62]. Delving into sexual attitudes and behaviour, E-type individuals and short sleep duration have been correlated with a tendency to be more promiscuous and to engage in unrestricted sociosexuality [63] or to exhibit lower levels of fidelity. In our study, we consider another sexual construct, the erotophobia–erotophilia dimension (willingness learned to respond to sexual stimuli along a bipolar continuum of affection and evaluation) [50]. To our knowledge, no previous studies have focused on the erotophobia–erotophilia dimension and chronotype. We found students with lower values in chronotype (E-type) showed a higher tendency to erotophilia. Interestingly, there were no differences in this dimension between students who used sexual protection methods and those who did not (*p* = 0.81). 

This investigation has certain limitations. Firstly, it is a cross-sectional study (coinciding with the period of vacations and exams), so our results did not allow us to determine the temporal direction of associations. Secondly, considering the non-response rate and the possible difficulty of completing the questionnaire, it is unknown whether our findings are representative of all Spanish university students. Thirdly, all the data are self-reported, so in the case of variables that can be objectively measured (for example, BMI), there may be variations. Future work should consider more direct physiological measures or use of diaries/journals to measure behaviour, and consider the social class of the students. Nonetheless, the findings of the present study were collected at two centres and were consistent with the existing literature. 

The strength of the manuscript focuses on the choice of variables (chronotype, Mediterranean diet, and sexual opinion), since to our knowledge, there is no scientific production that associates these variables. Another aspect of interest is the study population. 

## 5. Conclusions

Our results suggest eveningness was associated with lower adherence to the Mediterranean diet and a tendency towards erotophilia in a sample of university students. Chronotype was shown to be an important variable that interferes in two highly relevant areas at the university stage (diet and sexual opinion). Thus, chronotype must be considered among the measures employed to promote healthy habits (obesity prevention, sexual education, socialisation). The habits acquired at the university stage can determine later habits that determine the future lifestyle of each person. For this reason, it makes more sense to assess how schedules are programmed in universities and bear in mind that these schedules have an impact on the habits of students.

## Figures and Tables

**Table 1:** Sociodemographic variables and adherence to the Mediterranean diet (KIDMED).

Sociodemographic Variables	High Adherence *n* (%)	Medium Adherence *n* (%)	Low Adherence *n* (%)	*p*
Gender				
Male	55 (32.9)	71 (33.3)	25 (34.7)	0.964
Female	112 (67.1)	142 (66.7)	47 (65.3)	
University				
UCO	58 (34.7)	82 (38.5)	23 (31.9)	0.547
UCLM	109 (65.3)	131 (61.5)	49 (68.1)	
Place of residence				
University residence	15 (9.0)	22 (10.3)	4 (5.6)	0.05
Cohabit with parents	71 (42.5)	105 (49.3)	25 (34.7)	
Cohabit with peers	72 (43.1)	81 (38.0)	42 (58.3)	
Live with a partner	4 (2.4)	0 (0.0)	0 (0.0)	
Live alone	1 (1.4)	5 (2.3)	5 (3.0)	
Physical activity, mean (SD)	4465.26 (4486.99)	4629.54 (5181.39)	3675.68 (3722.09)	0.419
Erotophobia–erotophilia, mean (SD)	77.68 (21.27)	78.84 (19.27)	75.37 (18.02)	0.419

*n*, count; %, percentage; SD, standard deviation UCO: University of Cordoba; UCLM: University of Castilla-La Mancha. Chi-square test, Fisher’s test, and analysis of variance (ANOVA) were used to test differences.

**Table 2:** Chronotype and KIDMED score, index, and frequency of responses to each test item.

	M-Type (*n* = 76)	I-Type (*n* = 284)	E-Type (*n* = 92)	Total (*n* = 452)
**KIDMED index, % ** ^**1**^				
Low adherence	5 (6.6%)	43 (15.1%)	24 (26.1%)	72 (15.8%)
Medium adherence	34 (44.7%)	128 (45.1%)	51 (55.4%)	213 (46.6%)
High adherence	37 (48.7%)	113 (39.8%)	17 (18.5%)	167 (36.5%)
**KIDMED test (%, yes)**				
Takes fruit or fruit juice every day	58 (76.3%)	200 (69.9%)	49 (51.6%) **	307 (67.2%)
Has a second serving of fruit every day	36 (47.4%)	115 (40.2%)	24 (25.3%) **	175 (38.3%)
Has fresh or cooked vegetables regularly once a day	54 (71.1%)	175 (61.2%)	54 (56.8%)	283 (61.9%)
Has fresh or cooked vegetables more than once a day	54 (71.1%)	175 (61.2%)	54 (56.8%)	283 (61.9%)
Consumes fish regularly (at least 2–3 days/week)	46 (60.5%)	170 (59.4%)	47 (49.5%)	263 (57.5%)
Goes more than once a week to a fast-food (hamburger) restaurant	18 (23.7%)	79 (27.6%)	30 (31.6%)	127 (27.8%)
Likes pulses and eats them more than once a week	67 (88.2%)	226 (79.0%)	63 (66.3%) **	356 (77.9%)
Consumes pasta or rice almost every day (≥5 times/week)	24 (31.6%)	86 (30.1%)	34 (36.2%)	144 (31.5%)
Has cereals or cereal products (bread) for breakfast	61 (80.3%)	222 (77.6%)	63 (66.3%)	346 (75.7%)
Consumes nuts regularly (at least 2–3 times per week)	29 (38.2%)	97 (33.9%)	34 (35.8%)	160 (35.0%)
Uses olive oil at home	76 (100%)	281 (98.3%)	90 (94.7%) *	447 (97.8%)
Skips breakfast	7 (9.2%)	36 (12.6%)	31 (32.6%) **	74 (16.2%)
Has a dairy product for breakfast (yogurt, milk, etc.)	63 (82.9%)	225 (78.7%)	60 (63.8%) **	348 (76.1%)
Takes two yogurts and/or some cheese (40 g) daily	26 (34.2%)	104 (36.4%)	32 (33.7%)	162 (35.4%)
Has commercially baked goods or pastries for breakfast	20 (26.3%)	96 (33.7%)	29 (30.5%)	145 (31.7%)
Takes sweets and candy several times every day	7 (9.2%)	34 (11.9%)	15 (15.8%)	56 (12.3%)

M-type includes extreme and moderate morningness chronotype; I-type, intermediate chronotype; E-type includes extreme and moderate E-type. KIDMED, Mediterranean diet quality index for children and adolescents. * *p* < 0.05; ** *p* < 0.01. ^1^ Significant differences (*p* < 0.001) exist in the KIDMED index considering chronotypes.

**Table 3:** Associations between adherence to the Mediterranean diet, the erotophobia–erotophilia dimension, and chronotype.

	Chronotype				Erotophobia-erotophilia		
	β	95% CI	*p* ^a^		β	95% CI	*p* ^a^
Adherence to the Mediterranean diet	0.326	0.202, 0.449	<0.001	Adherence to the Mediterranean diet	0.600	−0.192, 1.392	0.137
BMI	0.057	−0.016, −0.130	0.126	BMI	0.407	−0.047, 0.861	0.078
Erotophobia–erotophilia dimension	−0.024	−0.04, −0.008	0.004	Chronotype	−0.916	−1.544, −0.289	0.004

BMI, body mass index; CI, confidence interval. Linear regression models were used to establish associations with continuous variables (adherence to the Mediterranean diet, BMI, and the erotophobia–erotophilia dimension). Lower values in chronotype are associated with E-type, whilst the highest values in the erotophobia–erotophilia dimension mean a tendency towards erotophobia. ^a^ Adjusted for age, sex, physical activity, and residency.
